# Bone mineral density and muscle mass in adults with developmental skeletal discrepancies

**DOI:** 10.1186/s12891-022-05538-9

**Published:** 2022-06-20

**Authors:** Reza Sharifi, Sheida Kordi, Farhad Noravesh, Yasaman Aghababaei, Majid Ramezani, Zhila Maghbooli

**Affiliations:** 1 Oral and Maxillofacial Surgery Department, Craniomaxillofacial Research Center, Tehran University of Medical Sciences, Tehran, Iran; 2 Oral and Maxillofacial Surgery Department, Dentistry School, Tehran University of Medical Sciences, Tehran, Iran; 3 Oral and Maxillofacial Surgery Department, Dental Branch, Craniomaxillofacial Research Center, Tehran Islamic Azad University of Medical Sciences, Tehran, Iran; 4 Faculty Dentistry School, Valencia University, Valencia, Spain; 5 Baqiyatallah University of Medical Sciences, Tehran, Iran; 6 Multiple Sclerosis Research Center, Neuroscience Institute, Tehran University of Medical Sciences, Tehran, Iran

**Keywords:** Osteoporosis, Muscle mass, Jaw deformity, Orthognathic surgery

## Abstract

**Background:**

It was aimed to investigate the musculoskeletal status in individuals diagnosed with skeletal discrepancies.

**Methods:**

This case–control study was performed on 35 patients with developmental skeletal discrepancies listed for orthognathic surgery as a case group and 33 patients who were nominated for wisdom tooth removal as a control group. All participants were aged 18–40 years and the research was carried out in the period between May 2018 and May 2019. Dual X-ray absorptiometry (DEXA) was used to assess bone mass density at three bone sites: total hip, femoral neck, and the spinal lumbar vertebrae (L1-L4). The appendicular muscle mass index (ASMI) was measured based on the four limbs from the DEXA scan.

**Results:**

Our data showed that 45.7% (16) of the case group were osteopenic or osteoporotic while in the control group only 21.2% (7) were osteopenic in at least one region (total hip, femoral neck, or lumbar) (*p*-value = 0.03).

Regarding muscle mass, there was significantly lower SMI in subjects with skeletal discrepancies (case group) compared with the control group (median (IQR) 5.9 (2.5) vs. 6.8 (2.9) (kg/m2), respectively, *p* = 0.04).

**Conclusions:**

There is an essential need for more studies to understand the exact interrelationship between musculoskeletal status and skeletal jaw discrepancies.

## Background

Osteoporosis and sarcopenia are two aging disorders whose economic impact matters to public health assessment. Both conditions are growing in intensity depending on lifestyle and life expectancy [1].

Sarcopenia is defined as a graduate drop in muscle mass [2]. In older adults, the quality and quantity of skeletal muscle are on a progressive decline, consequently reducing skeletal muscle strength and lowering metabolic function, and augmenting fatty connective tissues. Sarcopenic people are at risk of falls and osteoporotic bones remain at risk of fragility fractures. Osteosarcopenic patients suffer a remarkably higher mortality rate and pain with lower quality of life [3].

Osteoporosis is a disease that triggers alterations in bone density and structure, disrupting bone microarchitecture throughout life [1]. During the 2nd and 3rd decades of life, bone skeletal muscle mass shows the highest peak, although, from that age onwards, a progressive decline in bone and muscle mass is witnessed. Therefore, such phenomena are deemed as natural developments easy to diagnose and tackle at young ages, leading to a slowdown in the osteoporosis and sarcopenia processes [4].

The relationship between bone and muscle can be described by the mechanistic hypothesis, which illustrates the stimulation of bone mass to effectual osteogenesis through muscle contracture [5]. Studies using bone scans show that the bone density of any other part of the body is subject to change [6–9]. It is well known that mandibular shape can change by reducing skeletal bone density [10]. The mandibular bone density in individuals with a jaw discrepancy is likely an indicator of osteoporosis. Jaw discrepancies are, in fact, an imbalance between the size, shape, or position of the jaws relative to one other. The pathogenesis of a jaw discrepancy is not completely clear, but genetic and environmental factors seem to be involved in it. Recent studies have shown that mandibular bone density in individuals with such a discrepancy lacks adequate bone marrow quality, leading to even worse intraoperatively fractures [11].

One of the serious intraoperative complications of the bilateral sagittal split osteotomy (BSSO) in orthognathic surgery is a bad split or unfavorable fracture due to lower bone quality and bone marrow, especially in elderly patients and osteoporotic individuals [12]. Practically speaking, by assuming that in patients with skeletal discrepancies, the whole musculoskeletal status might be affected in a way that is in favor of a higher incidence of bad fractures, assessment of patients for diagnosis of osteoporosis can be useful.

It is likely patients with skeletal jaw discrepancies are at risk of alterations in musculoskeletal function. These patients undergoing orthognathic surgery are usually in the 2nd and 3rd decades of their lives, the best age to detect any abnormalities in total bone mass to prevent bone loss or muscle weakness.

The main purpose of this study was to investigate the musculoskeletal status in individuals diagnosed with skeletal discrepancies.

## Material and methods

This was a matched case–control study designed by the Tehran University of Medical Sciences. The participants of this study were individuals aged 18–40 who were enrolled from May 2018 to May 2019. As a case group, 35 patients were identified with developmental skeletal discrepancies. They were thereby nominated for orthognathic surgery and referred to the oral and maxillofacial department at Shariati Hospital.

Our control group consisted of 33 individuals, who were referred to Tehran University’s Dentistry Faculty, and were candidates for a wisdom tooth removal.

The exclusion criteria were a history of maxillofacial pathology, radiotherapy in the head and the neck region, corticosteroid consumption beyond three months, hyperparathyroidism, leukemia and multiple myeloma, chronic renal and hepatic disorders, vertebral or non-vertebral osteoporotic fractures, alcohol consumption, medications affecting bone metabolism, metabolic bone diseases, and bone metastases.

Demographic and clinical information including age, sex, status, history of systemic diseases, using calcium and vitamin D supplementations, sun exposure, and physical activity were obtained by a questionnaire. Nobody in the case and control groups had chronic disorders.

For sun exposure, a questionnaire was completed including 4 questions; time per day, day time, the average time of sun exposure, and using sunscreen during the last three months. Sun exposure was classified based on at least 10 min per day in the daytime (between 10 am to 3 pm).

For physical activity, the short format of the International Physical Activity Questionnaire (IPAQ) was used. Following IPAQ’s guidelines, frequency and duration of physical activity were converted to Metabolic Equivalent of Tasks. Physical activity was classified into two levels: inactive and active (moderate activity/health-enhancing physical activity).

### Defined developmental skeletal discrepancies

Based on the ANB angle, which is the most accurate and relevant diagnostic tool, all participants were categorized into three groups: Class I, Class II, and Class III [13]. Class I classification were recruited in the control group which means they had a relatively normal maxillomandibular relationship.

The basic radiographs were Panoramic, Lateral, and posteroanterior cephalometry. Study casts were assessed for the quantity of jaw discrepancy and asymmetry in the case group. All participants in the case group who were candidates for orthognathic surgery were classified as having an ANB angle of class II or III.

### Bone status measurement

Dual X-ray absorptiometry with a Lunar DPXMD densitometer (Lunar 7164, GE, and Madison, WI) was used to measure bone mass density and muscle volume at three bone sites: total hip, femoral neck, and lumbar spine vertebrae (L1-L4). Each person was categorized based on the World Health Organization (WHO) osteoporosis criteria: osteoporosis (T-score ≤ -2.5), osteopenia (-2.5 < T-score < -1), and normal (T-score ≥ -1).

### Muscle mass measurement

Muscle mass was estimated by dual energy X-ray absorptiometry (DEXA). The appendicular muscle mass index (ASMI) was calculated by the following equation: skeletal muscle mass (kg) divided by the square of height (m^2^).

### Statistical analysis

SPSS software (version 21) was used for data analysis. Nonparametric tests were used to compare variables between two groups; the Mann–Whitney U test for continuous variables and the Chi-square test or Fisher's exact test for categorical data. Numerical variables were expressed as the median (IQR) and categorical variables were presented as percentages (number). A Spearman’s correlation was used to determine the correlation between bone density and muscle mass. A *p*-value less than 0.05 was considered statistically significant.

## Results

Totally 68 patients were enrolled in the study; 35 in the case group and 33 in the control group. The two groups were not significantly different in terms of age (*P*-value = 0.1), sex (*P*-value = 0.3), and the body mass index (*P*-value = 0.5) (Table 1). There were not any significant differences in the prevalence of smoking, taking calcium and vitamin D supplementations, sun exposure, and physical activity (*p*-value > 0.05). The prevalence of obesity (> 30 kg/m^2^) was similar in the two groups; 5.7% in the case group and 6.1% in the control group (*p* = 0.9).

**Table 1:** Demographic characteristics and bone status in case and control group

	Case group (*N* = 35)	Control group (*N* = 33)	*P*-value
Age, year	27 (9)	29 (9)	0.1
Sex (Female)	71.4% (25)	60.6% (20)	0.3
BMI, kg/m^2^	23.8 (4.3)	23.4 (5.4)	0.5
Physical activity (activity)	2 (5.7%)	5 (15%)	0.2
Sun exposure at least 10 min (between 10^AM^-3^PM^)	22 (62.8%)	18 (54.4%)	0.4
Vitamin D and/or Calcium consumption	0 (0)	2 (6%)	0.2*
Smoking	0 (0)	1 (3%)	0.4*
**Bone Status**			
BMD total body (g/cm^2^)	1.06 (0.14)	1.09 (0.11)	0.6
T-score total body	-0.71 (1.50)	-0.70 (1.10)	0.4
BMD Hip (g/cm^2^)	0.90 (0.21)	0.97 (0.18)	0.2
T-score Hip	-0.20 (1.50)	0.00 (1.13)	0.2
BMD Femoral Neck (g/cm^2^)	0.81 (0.16)	0.82 (0.19)	0.4
T-score Femoral Neck	-0.70 (1.10)	-0.30 (1.18)	0.5
BMD Lumbar (L1-L4) (g/cm^2^)	0.99 (0.16)	1.01 (0.08)	0.2
T-score Lumbar (L1-L4)	-0.85 (1.45)	-0.20 (1.00)	0.1

Numerical variables were expressed as the median (IQR) and categorical variables were presented as percentages (number)

*BMD* Body Mass Density, *BMI* Body Mass Index, *L* Lumbar, *N* Number

*Fisher's exact test

### Bone and muscle mass status

The comparison of the BMD values, from the three regions (total Hip, femoral neck, and lumbar spine (L1-L4), showed no significant differences between the case and control groups (*p*-value > 0.05) (Table 1). While in terms of osteoporosis, the prevalence of osteopenia or osteoporosis (at least in one bone site) was higher in the case group compared with the control group (45.7% vs. 21.2%, respectively, *p* = 0.03).

Only one person in the case group had osteoporosis while there were no osteoporotic patients in the control group. In terms of muscle mass, the ASMI was lower in the case group compared with the control group; the median (IQR) 5.9 (2.5) vs. 6.8 (2.9) (kg/m^2^), respectively, *p* = 0.04. In the case group, there was a significant positive correlation between muscle mass (ASMI) and bone density in the hip site (BMD Hip: rho = 0.5, T-score hip: rho = 0.4, *p* < 0.01) in women while there was not any significant correlation between muscle mass and bone density in men (*P* > 0.05).

In the control group, there was a significant correlation between ASMI and total BMD (rho = 0.6, *p* = 0.02) but no other sites. In women, there was not any correlation between ASMI and total BMD or other specific sites (Hip, femoral neck, and lumbar sites) (*p* > 0.05).

## Discussion

Our finding has shown that patients with skeletal discrepancies are at more risk of osteopenia and lower muscle mass compared with control people of the same age.

Although no study has comprehensively assessed the musculoskeletal status of the whole body and specific bone sites in patients with developmental skeletal jaw discrepancies, Konstantynowicz J et al. considered the total bone density and lumbar spine, hip, and head sites in the adolescence group. They reported that reduced total bone mineral density is associated with dental malocclusion in only men at 14–18 years old [14].

The case group of our population study was patients who were candidates for orthognathic surgery with a jaw discrepancy as well as dental malocclusion. Over 60% of them were in the second and third decays of their life and it is expected to have the maximum bone and muscle mass of their life.

However, these patients experience prompt weight loss during 4 weeks postoperative period because of the inability of mastication and intermaxillary fixation (IMF) [15]. It has been suggested weight loss in early adulthood can be a risk factor for lower bone mineral density (BMD) and an increase in osteoporosis in later life [16]. It is of importance for surgeons to consider these risk factors to improve the management of the patients specifically just following the surgery.

In the field of research, previous studies considered the relationship between maxillofacial morphology, bone characteristics, and bone metabolic markers in patients with jaw deformity [17]. Saito et al. (2015) showed a higher level of deoxypyridinoline as a collagen mature degradation marker and tartrate-resistant acid phosphatase isoform 5b (TRACP-5b) as an osteoclast marker in patients with skeletal discrepancy of Class III [18].

In another study, Taguchi et al. estimated bone status based on mandibular cortex erosion through evaluation of routine dental radiographs. The authors have reported the significant association between mandibular cortex erosion and bone turnover markers such as N-telopeptide (NTx), Cross-links of type I collagen, and alkaline phosphatase (ALP), which are increased in osteoporosis. As NTx is a bone marker of higher susceptibility when compared to ALP, it can serve as a useful indicator in detecting early stages of osteoporosis among postmenopausal women [19].

Higher levels of bone resorption markers have been found in lower BMD and deterioration of bone microstructure, which can be a prognostic factor for osteoporosis in the future. Furthermore, combining diagnostic tools of BMD measurement and bone resorption markers has proven to be more valuable in predicting the risk factors of osteoporosis in patients suffering from skeletal jaw discrepancies. In the current research, we didn’t measure circulating levels of bone markers or bone markers in bone tissue in our study population.

Bone mass density is regulated when the process of bone formation by osteoblasts is boosted while osteoclasts cause a decrease or degeneration. All concerns about assessing the dynamics of bone tissue during puberty are based on the fact that the stage plays a crucial role in optimizing skeleton strength and minimizing potential bone resorption [17]. Also, early-stage osteoporosis can be diagnosed in advance thus helping eliminate excessive osteoporosis-related medical costs and prevent pathologic fractures in patients with skeletal jaw discrepancies during orthognathic surgery or secondary during a lifetime.

Our data also showed there was a significant correlation between muscle mass and bone status in hip and femoral neck regions in women (not men) with a jaw discrepancy. The relationship between muscle mass and bone density is well established. Our findings suggest that young women with a jaw discrepancy are likely more at risk of both muscle weakness and osteoporosis before age of menopausal. Further studies need to be designed and consider this possibility.

In the present study, some limitations in our study are worth noting. Firstly, the design of our study is matched case control. So, we cannot explain the cause and effect relationship between low muscle mass and bone density, and skeletal jaw discrepancies. Designing large-scale studies need to evaluate the risk association between them.

Secondly, some lifestyle-related factors could have an impact on muscle mass and bone status. To minimize the effects of lifestyle, we considered smoking habits, sun exposure, physical activity, and vitamin D and calcium supplementations. There were not any significant differences between the two groups. Thirdly, we measured the bone density of three skeletal regions, the total hip, femoral neck, and the spinal lumbar vertebrae (L1-L4). Panoramic radiographs or other methods may help in considering the bone status of the jaw. Of note, we used DEXA as a gold standard for assessing bone and muscle mass.

Since no study in the literature has been reported to clarify the status of bone markers in patients with skeletal discrepancies, more efforts in this scope of research need to comprehend this interrelationship. Finally, it has been suggested that the muscle mass might be affected by tooth loss [20]. We did not collect data of dental problems including tooth loss, dental hygiene, etc.

In conclusion, our study suggests that patients with skeletal discrepancies are at risk of low muscle mass and bone density. Considering skeletal-muscle status in patients with skeletal discrepancies the early diagnosis of osteoporosis or muscle weakness can diminish the complications of severe osteoporosis in later life. Also, it is important to predict and prevention of unfavorable fractures in osteotomy sites of the mandible and very rarely in the maxilla during orthognathic surgery in patients with skeletal discrepancies.

## Data Availability

The data that support the findings of this study are available from the Research Deputy of Tehran University of Medical Science but restrictions apply to the availability of these data, which were used under license for the current study, and so are not publicly available. Data are however available from the corresponding authors upon reasonable request and with permission of the Research Deputy of Tehran University of Medical Science.

## Abbreviations

ALP: Alkaline phosphatase
ASMI: Appendicular muscle mass index
BMD: Bone mineral density
BMI: Body mass index
DEXA: Dual X-ray absorptiometry
IPAQ: International Physical Activity Questionnaire
NTx: N-telopeptide
TRACP-5b: Tartrate-resistant acid phosphatase isoform 5b
